# Development and validation of genomic predictors of radiation sensitivity using preclinical data

**DOI:** 10.1186/s12885-021-08652-4

**Published:** 2021-08-20

**Authors:** Venkata S. K. Manem

**Affiliations:** 1 Quebec Heart & Lung Institute Research Center, Quebec City, Quebec G1V 4G5 Canada; 2 Faculty of Pharmacy, Laval University, Quebec City, Quebec G1V 0A6 Canada

**Keywords:** Radiogenomics, Biomarkers, Cancer genomics, Machine learning, Personalized medicine

## Abstract

**Background:**

Radiation therapy is among the most effective and commonly used therapeutic modalities of cancer treatments in current clinical practice. The fundamental paradigm that has guided radiotherapeutic regimens are ‘one-size-fits-all’, which are not in line with the dogma of precision medicine. While there were efforts to build radioresponse signatures using OMICS data, their ability to accurately predict in patients is still limited.

**Methods:**

We proposed to integrate two large-scale radiogenomics datasets consisting of 511 with 23 tissues and 60 cancer cell lines with 9 tissues to build and validate radiation response biomarkers. We used intrinsic radiation sensitivity, i.e., surviving fraction of cells (SF2) as the radiation response indicator. Gene set enrichment analysis was used to examine the biological determinants driving SF2. Using SF2 as a continuous variable, we used five different approaches, univariate, rank gene ensemble, rank gene multivariate, mRMR and elasticNet to build genomic predictors of radiation response through a cross-validation framework.

**Results:**

Through the pathway analysis, we found 159 pathways to be statistically significant, out of which 54 and 105 were positively and negatively enriched with SF2. More importantly, we found cell cycle and repair pathways to be enriched with SF2, which are inline with the fundamental aspects of radiation biology. With regards to the radiation response gene signature, we found that all multivariate models outperformed the univariate model with a ranking based approach performing well compared to other models, indicating complex biological processes underpinning radiation response.

**Conclusion:**

To summarize, we found biological processes underpinning SF2 and systematically compared different machine learning approaches to develop and validate predictors of radiation response. With more patient data available in the future, the clinical value of these biomarkers can be assessed that would allow for personalization of radiotherapy.

## Background

Radiotherapy is the mainstay of treatment for most types of cancers [1]. Clinically, it is known that over half of all cancer patients undergo radiotherapy either as a primary therapeutic regimen or in an adjuvant or a neoadjuvant setting. Current radiotherapy regimens are largely ‘one-size-fits-all’ and do not allow for patient-specific individualization [2]. In recent years, we have witnessed a rapid evolution of technological advancements in radiotherapy treatment delivery and dose conformity through particle therapies, image-guided techniques [3, 4] and the latest addition being the flash radiotherapy technique [5]. These significant advances have allowed the adaptation of radiation treatment planning fields based on anatomical changes of the gross tumor volume (in maximizing tumor control probability [6] and minimizing late toxicities either alone [7, 8] or in combination with chemotherapy [9]). However, designing personalized radiotherapeutic regimens based on the tumor’s biological features is still hindered by the lack of appropriate clinical biomarkers [10]. It is well known that radiotherapy techniques deliver a known physical dose with a high degree of accuracy to similar tumors, however the radiation efficacy varies widely due to the inter-patient variability [11, 12] and intra-tumor heterogeneity [13]. To tailor radiation therapy to individual patients, it is important to build predictive assays that can potentially be used to design genomically-driven dosing, facilitating decision making between treatments such as neoadjuvant or adjuvant radiotherapy or chemo-radiotherapy. This could augment the existing radiobiological therapeutic strategies to genomically-driven personalized radiation regimens. Along these lines, the application of transcriptomic fingerprinting to administer OMICS-driven therapies is making inroads to pre-clinical and clinical settings in precision radiation oncology [14].

Access to OMICS data (such as, transcriptomics, proteomics, epigenomics, etc.) has led to the emergence of data-driven approaches to better understand the biological factors that influence tumor sensitivity response to various therapies. Many physical and biological factors are known to influence tumor response to irradiation that include total radiation dose [15], hypoxia [16], reoxygenation [17], tumor doubling time, fractionation scheme [18] and intrinsic radiosensitivity [16]. The continuous inflow of transcriptomics data holds great promise to develop novel molecular biomarkers of radiation response. Several groups have conducted comprehensive gene expression profiling and built genomic predictors of radiation sensitivity under both oxic and hypoxic conditions using cancer cell lines and patient data [10].

Mainly, two sets of approaches have been applied to predict the radiation sensitivity, namely, bottom-up (data-driven) methods and top-down (hypothesis-based) methods. A comprehensive set of various techniques applied to build radiation response gene signatures can be found in a recently published work by Manem et al. [10]. All the genomic signatures of radiation sensitivity were built using the NCI-60 panel dataset with limited to no independent external validation raising concerns about their reproducibility. Some of the reasons include noise in the cell line data and relatively low number of samples compared to the number of predictors. Recently, Yard et al. published a large radiogenomic dataset of unprecedented size consisting of 511 cancer cell lines, which were subjected to different types of high-throughput screening. One of them is a radiation sensitivity screen where radiation was administered to the cell lines to see how well they inhibit the growth to obtain radiation response data. Concurrently, the same cancer cell lines were profiled at the transcriptomic level with the goal of trying to correlate the molecular features of these cells with radioresponse allowing for generation of predictive models. Till date, none of the studies in the literature have utilized these two radiogenomics datasets in developing and validating radiation response biomarkers. Given the complexity of the radiation response predictions and the risk of discovering biological artifacts, there is a dire need to combine these two large-scale radiogenomic datasets and build robust and reproducible multivariate genomic predictors and validate them on fully independent datasets. There has been a paucity of studies that systematically explored various modelling approaches based on data from the two largest radiation cell line screens. With the overall goal to improve the accuracy of radiation sensitivity predictions, we attempt to address these gaps in the current work.

In this study, we used two large-scale radiation genomic datasets and compared different machine learning approaches to predict radiation sensitivity. To the best of our knowledge, *this is the first time that both these datasets were analyzed in a single study, which should provide us with enough sample size to build robust genomic predictors and validate them in a fully independent dataset (discovery dataset = 511 cancer cell lines and validation dataset = 60 cancer cell lines)*. The generation of molecular predictors of radiation response in the preclinical setting like the models we validated in this study can be incorporated into the design of clinical trials. This can potentially accelerate the emergence of biologically-driven radiation regimens based on the genomic characteristics of an individual’s tumor.

## Methods

To develop robust molecular predictors of radiation response, we used the recently published by Yard et al., termed as *Cleveland data set* (CL) and Amundson et al., termed as NCI-60 (NCI). We collected, curated and annotated these two large-scale radiogenomics datasets using our recently published *RadioGx*, a computational platform to perform radiogenomic analyses [19]. The CL dataset consists of 511 cancer cell lines spanning across 23 histologies, while the NCI dataset has 60 cell lines spanning across 9 histologies. This large compendium of datasets will be used to build and validate genomic predictors of radiation sensitivity.

### Radiation dose response data

In the CL dataset, multiple radiation doses ({1Gy, 2Gy, 3Gy, 4Gy, 6Gy, 8Gy}) were administered to obtain the dose response data, while in the NCI dataset only three radiation doses of {2Gy, 4Gy, 6Gy} were administered. Through this radiation dose response data, we can obtain summary indicators that will be useful for preclinical investigations. Radiation sensitivity can be described by the area under the curve (AUC) of the fitted radiobiological model to the dose response data or at a specific dose level such as surviving fraction at 2 Gy (SF2). Although both AUC and SF2 have frequently been used in the literature, there is currently no consensus regarding the optimal indicator for use across studies when probing the biological determinants of radioresponse. However, the conventional indicator to determine intrinsic radiation sensitivity is the surviving fraction at 2 Gy (SF2) measured by clonogenic survival assays. This has been supported by ex vivo studies that demonstrated that tumor control probability may be associated with SF2 following radiotherapy treatment [20]. In the current study, we will use SF2 as the indicator of radiation response to build and validate the genomic predictors of radiation sensitivity.

### Gene expression data

Raw Illumina RNA-seq profiles of the CL dataset were retrieved from the CCLE website (http://www.broadinstitute.org/ccle/) and for the NCI dataset, we retrieved the gene expression profiles using the R package, *rcell miner*. This data has earlier been processed using our *RadioGx* platform [19]. Analyses was restricted to the genes common to the CL and NCI datasets, for a total of 12,258 genes.

### Modelling approaches

To build genomic predictors of radiation sensitivity, we used the following regression-based linear methods as described below -

1. Single gene: Gene that is strongly correlated with SF2 was computed using the Spearman correlation. This gene was then used to fit a univariate regression model. This was the simplest approach and will serve as a benchmark for multivariate models.
2. Rank gene ensemble: The most significant genes were selected based on the ranking of correlation between SF2 and gene expression. These genes were then combined with their respective univariate regression models using an ensemble approach. The simplest ensemble approach was done by aggregating the predictions obtained from each univariate model.
3. Rank gene multivariate: This approach was similar to the rank gene ensemble method, except that the most significant genes were used to fit a multivariate regression model.
4. mRMR: Two feature selection techniques were employed that use minimum redundancy and maximum relevance technique to select genes that were most relevant and non-redundant through exhaustive and bootstrap methods [21]. The exhaustive method initializes multiple feature selection procedures, and *K* mRMR solutions were produced in which the first selected feature was guaranteed to be different. While the bootstrap method resamples the original dataset with replacement to generate k bootstraps, and classical mRMR feature selection was performed for each of the bootstrapped datasets, thus generating *K* mRMR solutions. These genes are then used to fit in a multivariate regression model.
5. ElasticNet: This is a widely used regularized multivariate regression technique with L1/L2 penalty that is optimized by a 10-fold cross-validation. We fixed the value of α = 0.5, and selected the optimal regularization parameter λ = e^γ^, where γ ε (− 6, 5) (taken from drug response modeling done on cell lines [22]), through optimizing the mean squared error of the model in inner cross-validation.

Although the ‘rank gene ensemble’ and ‘rank gene multivariate’ are multivariate by nature, they do not take into consideration the redundancy in features. Hence, we implemented the mRMR feature selection, as it selects genes that are strongly correlated with SF2 while minimizing their redundancy. As carried out in previous comparative studies, in order to facilitate the comparison between different modelling strategies, a 30 gene signature has been demonstrated to be a trade-off between the modelling complexity and its relevance [23]. Hence, we fixed the number of features to 30 for the ‘rank gene ensemble’, ‘rank gene multivariate’ and ‘mRMR’ approaches.

### Model performance

The performance of each model was evaluated using the concordance index metric, which is a generalization of the area under the ROC curve. The concordance index is defined as the probability that two variables will rank a random pair of samples in the same order. A random predictive would result in an index of 0.5, while a perfect predictor yields an index of 1.

### Analysis framework

**Fig. 1:**
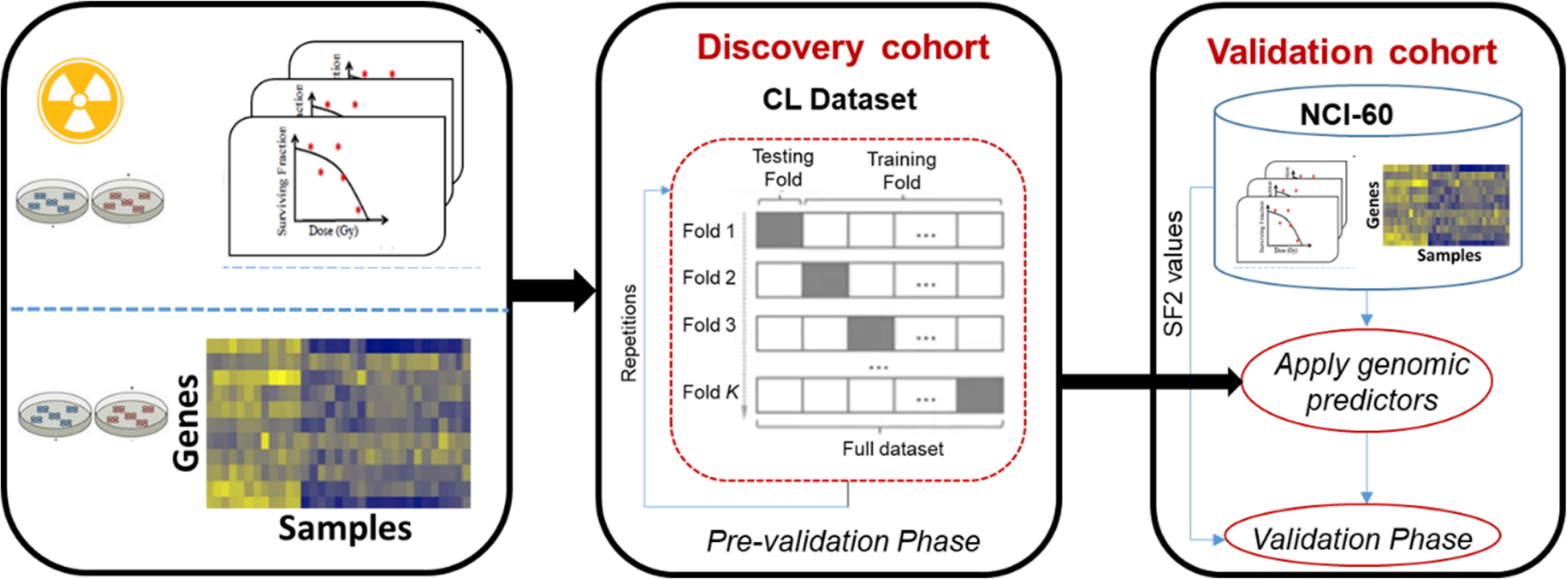
Analysis pipeline of the study. We first performed the pre-validation analysis of genomic predictors for radiation sensitivity using the cross-validation framework in the discovery cohort, *CL* dataset. Genomic predictors using the full training set are then built and evaluated their performance in a fully independent validation dataset, NCI-60 cohort

The analysis pipeline is illustrated in Fig. 1. The CL dataset was used as the discovery cohort, while the NCI dataset was used as the validation cohort. We first carried out the pre-validation phase on the discovery dataset that consisted of 10 iterations/repetitions of 10-fold cross-validation for each of the models presented above. We then trained each of the models on the CL dataset, tested them on the NCI dataset and calculated the accuracy using the concordance index. This was repeated ten times to obtain an average value of accuracy for each model.

### Biological determinants of SF2

The pathway enrichment analysis on the gene expression profiles was carried out using the gene set enrichment analysis (GSEA) method [24] with pathways defined by the ‘REACTOME’ database consisting of 1498 pathways (downloaded from MSIGDB). Genes were ranked based on their coefficient of correlation between the gene expressions and the radiation response variable, SF2. GSEA was then used to compute the enrichment score for each pathway with statistical significance calculated using a permutation test (10,000 permutations) as implemented in the piano package [25]. Nominal *p*-values obtained for each pathway were corrected for multiple testing using the false discovery approach (FDR).

## Results

**Fig. 2:**
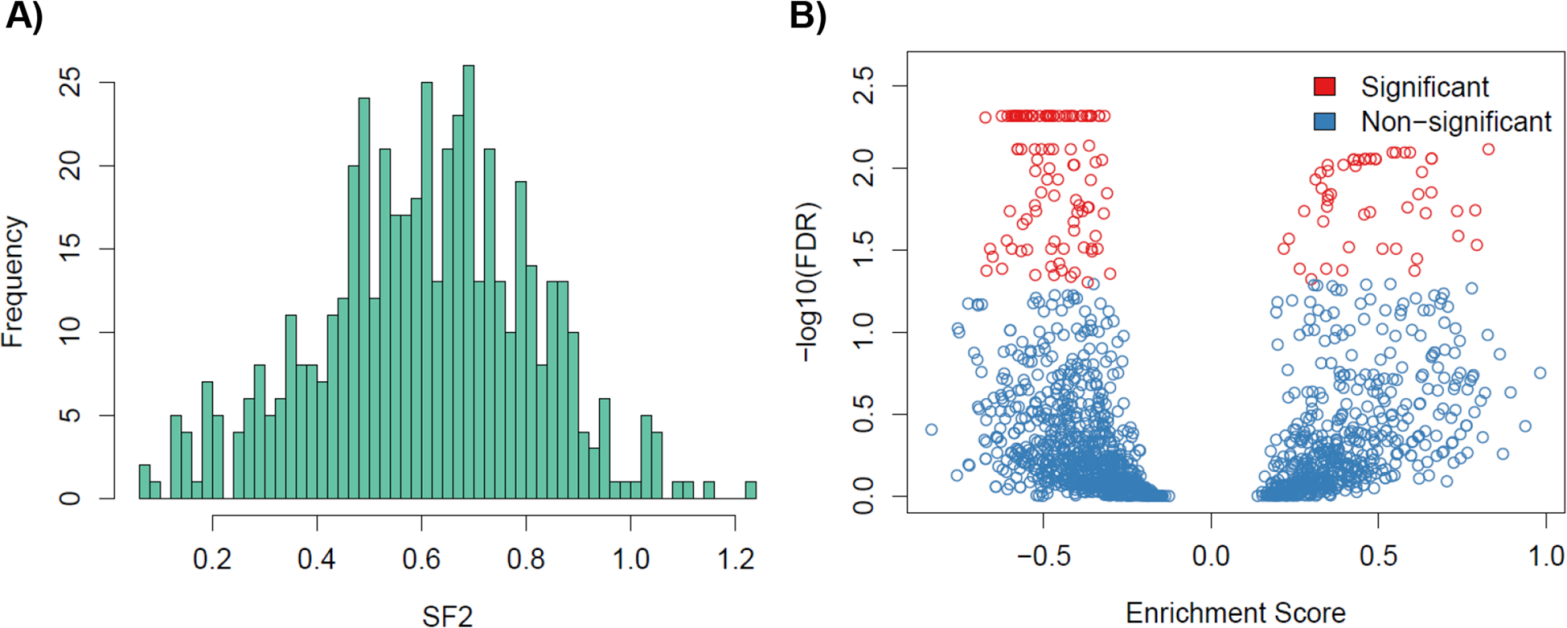
Biological determinants of radiation sensitivity. **A)** Distribution of SF2: Histogram of SF2 values with 511 cancer cell lines in the CL dataset. **B**) Biological processes underpinning SF2: FDR for each molecular pathway illustrating the statistical significance and enrichment score

To assess differences in the radiation response distribution across all profiled cancer cell lines in the largest dataset (CL cohort), we plotted the probability density distribution of SF2 values (Fig. 2A). We observed a range of radiation response profiles with mean value of 0.6 (SD = 0.2).

To investigate the molecular processes that drive SF2, we performed pathway analysis on the largest dataset consisting of 511 cancer cell lines. For an FDR < 5%, we found a total of 159 molecular pathways that were enriched with SF2 (Fig. 2B). Out of which, 54 pathways were positively enriched and 105 pathways negatively enriched. Importantly, we found two categories of biological processes that were enriched, namely, cell cycle and repair pathways. It is well known that cell cycle progression post irradiation is a known factor to determine cell survival or radiation-induced cell death. We found three cell cycle pathways to be significantly enriched - REACTOME CELL CYCLE, REACTOME CELL CYCLE CHECKPOINTS, and REACTOME CELL CYCLE MITOTIC. DNA repair is a crucial component for cell survival post irradiation. Among the DNA repair pathways, we found REACTOME NUCLEOTIDE EXCISION REPAIR, REACTOME DNA REPAIR, REACTOME SUMOYLATION OF DNA DAMAGE RESPONSE AND REPAIR PROTEINS, REACTOME DNA DOUBLE STRAND BREAK REPAIR, REACTOME HOMOLOGY DIRECTED REPAIR, REACTOME GLOBAL GENOME NUCLEOTIDE EXCISION REPAIR [26]. To summarize, we characterized the biological determinants underpinning SF2, supporting the biological relevance of these pathways in the context of radiation therapy.

### Comparison of models

**Fig. 3:**
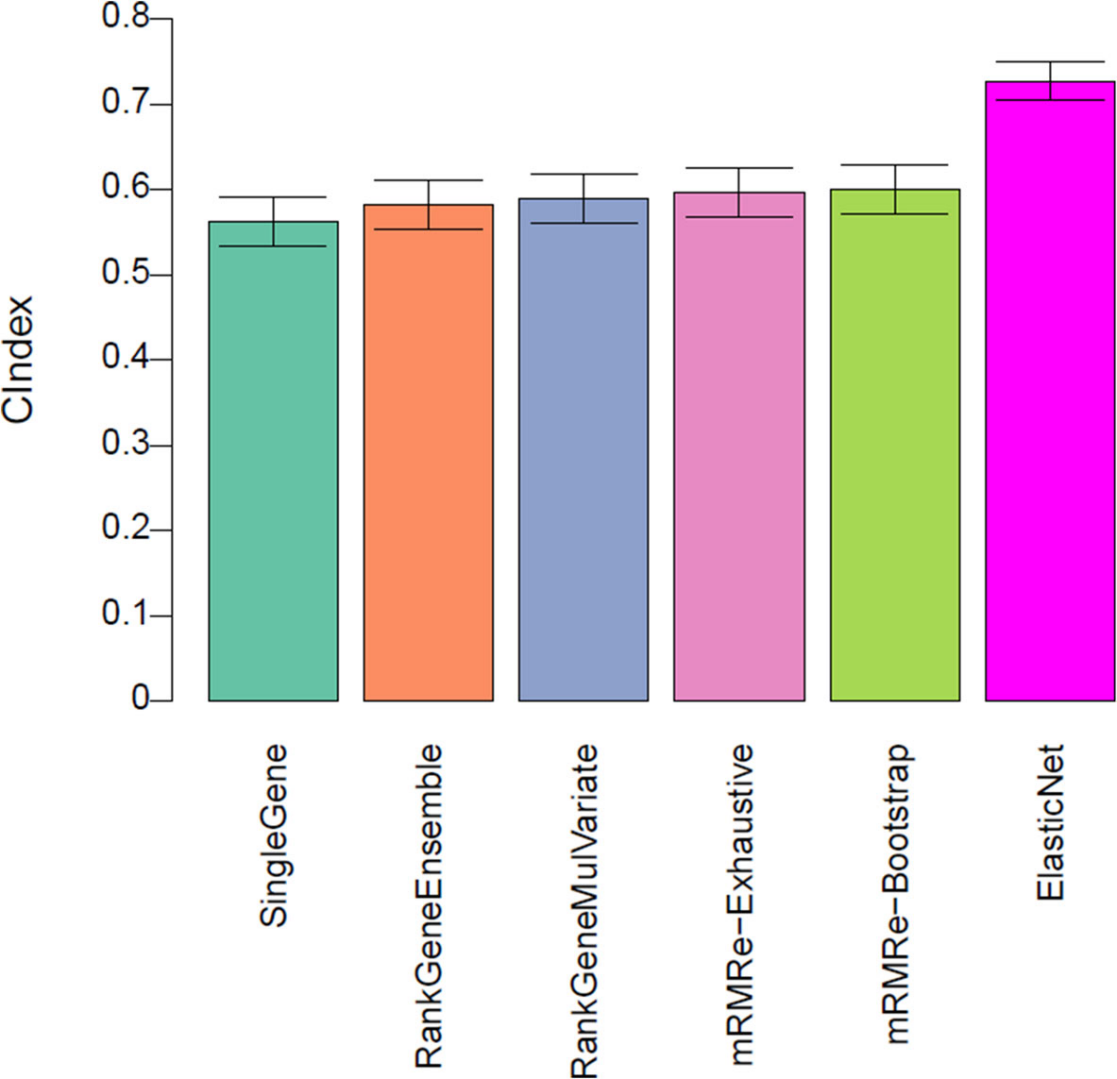
Model performance of genomic predictors of radiation sensitivity in the pre-validation phase. The prediction performance of the five models assessed by concordance index between the predicted and observed SF2 values. Predictions were averaged in 10 iterations of 10-fold cross-validation in the discovery dataset (CL). The error bars represent the 95% confidence interval of the performance computed during the 10 repetitions of cross-validation

We compared the five genomic predictive models in the discovery dataset (CL) using a cross-validation strategy consisting of 10 iterations of 10-fold cross-validations. The basal gene expression profiles and SF2 of all cell lines in the training set were used to identify genes that were strongly associated with radiation response. To reduce the dimensionality of feature space, we selected 1000 genes exhibiting the highest variance in the discovery cohort. We used six machine learning methods (with two techniques from mRMR) to construct predictive models from gene expression profiles. The performance of the models is presented in Fig. 2. As can be seen in Fig. 3, we observed a relatively good performance for multivariate models with concordance index value close to 0.6 and above. The performance of the univariate model (single gene) was poor. Multivariate models based on mRMR yielded the same performance irrespective of the two techniques (exhaustive and bootstrap). Overall, Elasticnet resulted in a concordance index of greater than 0.6.

**Fig. 4:**
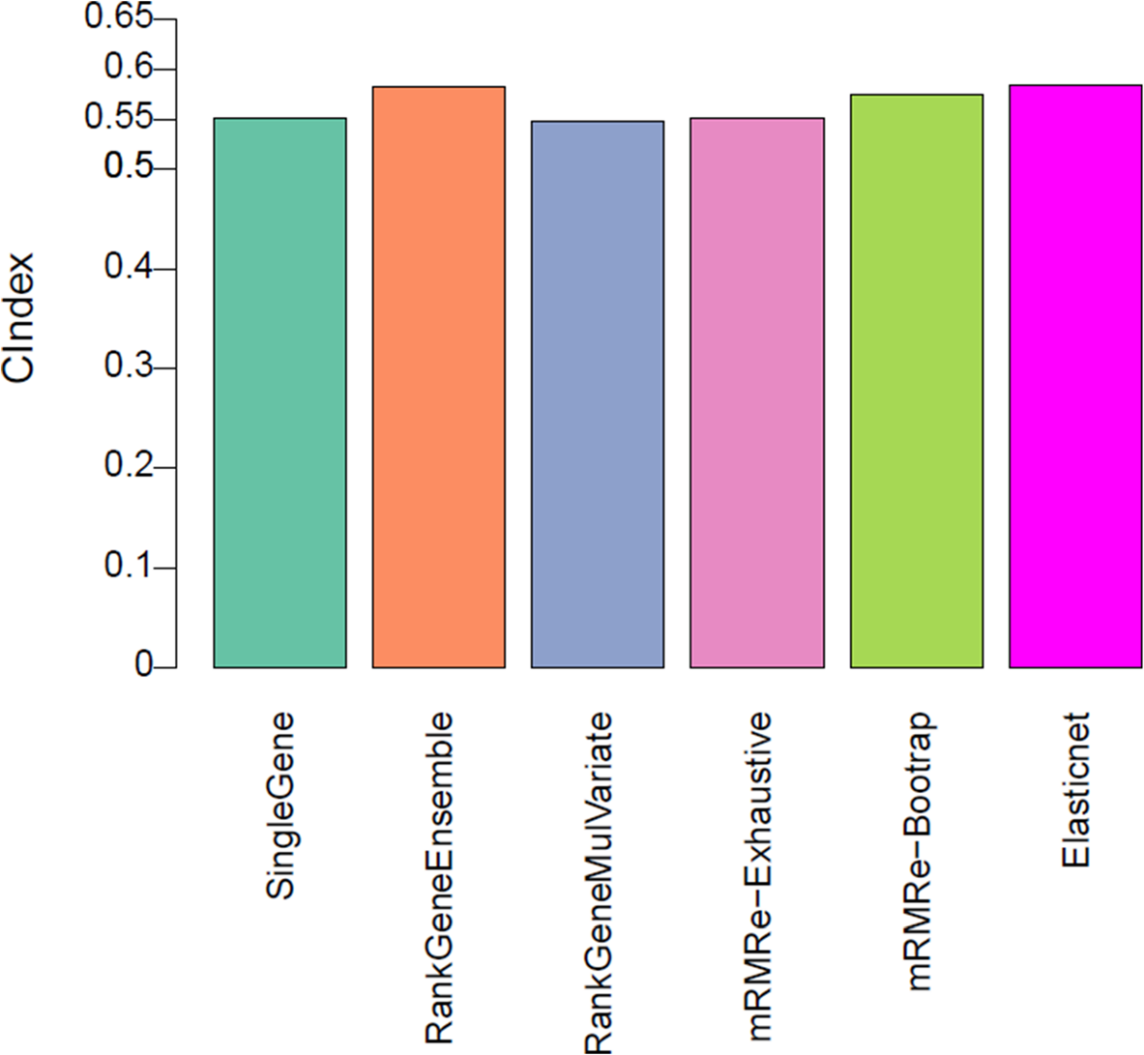
Model performance of genomic predictors of radiation sensitivity in validation data. The prediction performance of the five models assessed by concordance index between the predicted and observed SF2 values in the NCI dataset

We further validated the performance of the five genomic models in the NCI data, which is a fully independent validation dataset. This is the most challenging as it allows us to address whether the developed genomic predictors are generalizable to new datasets. As presented in Fig. 4, the performance of the models (single gene, rank gene multivariate and mRMR) predictive of SF2 was close to what was estimated in the pre-validation phase. Although ElasticNet performed well in the pre-validation phase, however, its performance decreased on the new samples. Overall, rank gene ensemble performed well in the validation dataset compared to other models (with a concordance index of 0.59).

## Discussion

Precision medicine has generally been drug-based using preclinical model systems and very little attention has been paid to the discipline of radiation oncology. In the last few years, radiation genomics has emerged as a new research field, which can help investigate changes in the transcriptome induced by radiotherapy as well as develop predictive biomarkers of radiation response. This has spurred research towards building OMICS-driven biomarkers using gene expression profiles from in-vitro or cell line data [19].

Despite the growing field of biomarker research in radiotherapy, none of these predictive biomarkers have been translated to routine clinical use. Translation of preclinical-based omics biomarkers to the standard of care requires a rigorous model development and validation process. Along these lines, the Institute of Medicine provided recommendations comprising clinical utility to regulatory issues on OMICS-based methods. Lisa McShane et al. developed a list of 30 criteria for omics-based assays that were broadly classified into the following components: specimen and assay issues, model development, performance evaluation, clinical trial design, ethical, legal and regulatory issues [27]. With these criteria in mind, evaluating the readiness of biomarkers for standard of care would be a major advance to measure a patient’s radiation sensitivity allowing for personalized treatments, particularly in: a) reducing the total radiation dose for radiation sensitive patients; b) escalating the dose for radiation resistant patients; and, c) increasing the efficacy with chemotherapy (radiation sensitizing compounds) in radiation resistant patients.

Several efforts have been directed towards developing radiation sensitivity gene signatures using cell data obtained from clonogenic survival assays [10]. Almost all of the studies in the literature developed radiosensitivity gene signatures using the NCI-60 panel and demonstrated poor predictive performance on external validation datasets. One of the reasons is that the number of cell lines used in these published studies were insufficient to develop robust predictors. Hence, none of these signatures have been translated to clinical use, highlighting the need to build robust and reproducible biomarkers of radiation response for future interventional studies [14].

Development and validation of robust biomarkers will benefit from expanding the number of cancer cell lines for which gene expression data and dose response data are available. Along these lines, Yard et al. recently published a large radiogenomic dataset of unprecedented size, including 511 cancer cell lines, in which each of the cell lines was screened for cell viability and gene expression profiles. While these two datasets were available in the literature, none of the studies have analyzed them together to build robust genomic predictors. We utilized this opportunity and combined the datasets generated from the two largest studies to build and validate robust gene expression predictors of radiation response.

The main objective of our study was to *i)* investigate the molecular processes underpinning radiation response using SF2; and *ii)* compare different modeling approaches to build genomic predictors of radiation response. It is beyond the scope of this study to identify which would be the best model to measure radiation sensitivity. Radiosensitivity can be measured as the surviving fraction of cells at 2Gy (SF2) of radiation dose. SF2 is considered to be the gold standard and is supported by strong clinical evidence. Moreover, several studies have also shown that in vitro measurements of SF2 were associated with in-vivo radioresponse as well. Based on this, we considered SF2 as the radiation response variable or the outcome of interest.

We performed pathway analysis using the GSEA method and obtained 159 statistically significant pathways. Among these pathways, we found cell cycle and DNA repair pathways to be enriched, which are aligned with the fundamental aspects of radiation biology. These findings facilitate a potential understanding for the biological mechanisms behind the association of radiation response at transcriptomic level, and eventually may lead to a more mechanistically derived gene signature. For the comparative study of machine learning approaches to build predictors, we implemented four different multivariate models (rank gene ensemble, rank gene multivariate, minimum redundancy and maximum relevance and ElasticNet) and one univariate model (single gene) with a total of five different modelling approaches. Due to the availability of two datasets, we used the CL dataset as the discovery cohort (*n* = 511 cancer cell lines) and NCI dataset as the validation cohort (*n* = 60 cancer cell lines). We found that all the multivariate models outperformed the univariate model, while ElasticNet showed a significant gain of predictive power compared to other models in the pre-validation phase. On the contrary, applying these models in the validation cohort indicated that rank gene ensemble approach to be a better model with higher concordance index compared to other models.

Through our previous works, we characterized the biological imprecision of one-size-fits-all radiation dosing regimens by assessing patient-specific radiation-induced toxicities [6, 8]. Furthermore, we curated a repertoire of published radiation response gene signatures [10], and reported that all the genomic signatures of radiation sensitivity were built using the NCI-60 dataset with limited to no independent external validation raising concerns about their reproducibility. This has spurred the need to develop pre-clinical radiotherapeutic discovery pipeline to build biomarkers. To achieve this, we build a unique computational platform, RadioGx [19], that could be accelerate pre-clinical research in radiation medicine. The current study builds upon our recent work of RadioGx, showcasing the utility of the platform by integrating two largest radiogenomic datasets to develop robust biomarkers of radiation response, which is a first modeling effort in this field. The novelty of this work stems from integrating two largest independent datasets to build pre-clinical biomarkers through comparing various modeling approaches. We obtained reasonably accurate predictions that will advance our approaches to build robust radiation response biomarkers through integrating large-scale radiogenomics datasets in the future. Genomic predictors built in this study have shown promising performance in independent preclinical datasets, which is a step towards assessing their clinical relevance on patient data. Once successful, these predictors could be used to identify radiation sensitive and radiation resistant populations, thereby, improving the therapeutic efficacy of radiotherapy. More importantly, with the availability of pre-clinical and patient OMICS data in the future, it will be possible to validate genomic predictors on those cohorts and identify groups of samples in which the association of radiation sensitivity with genomic features are transferable from cell lines to patients and samples in which they are not. This can potentially accelerate the emergence of biologically-driven radiation biomarkers based on the genomic characteristics of an individual’s tumor.

Our study could be extended in a couple of ways to improve the accuracy of model predictions with gene expression data through the following means: *i)* One of the challenges in the statistical analyses of biological data include assay types, complexity of experimental designs along with the non-standard distributions of measured data that result in noisy measurements. This is especially true for high-throughput radiation cell line screening studies. Through replicate measurements under the same cell culture conditions, it is feasible to measure the degree of noise for a given experimental protocol and assay. Therefore, introducing new statistical approaches to measure noisy biological data could potentially improve the model predictions; *ii)* Exploring the sensitivity of radiation response variables such as AUC (area under the curve of the fitted radiobiological model) and 1-AUC could shed light into the choice of a radiation response characterization metric on the accuracy of predictions.

## Conclusion

Precision medicine has generally been driven by drug-based approaches and has eluded the field of radiation oncology. Availability of OMICS data has made it possible to build genomically-driven approaches to prescribe personalized radiation therapeutic regimens. The application of transcriptomic fingerprinting to build biomarkers of radiation sensitivity are at the horizon and require rigorous pre-clinical assessment before translating them to the standard of care. Current genomics-based radiation sensitivity biomarkers have shown limited external validation raising concerns about their reproducibility. Based on this premise, in our current study we were able to build genomic predictors by integrating two large-scale radiation genomics datasets, which is the first of a kind of effort in this field. The genomics predictors built in this study have shown promise, however we need to assess their clinical relevance in other pre-clinical and patient data sets (when available) before building a predictive assay for clinical setting. As radiation therapy is a commonly administered therapeutic modality, clinically validated radiation sensitivity biomarkers will have a great potential to improve the treatment outcomes, which could eventually have a huge impact on radiation oncology practice.

## Data Availability

The dataset(s) supporting the analysis of this article are available in the *RadioGx* repository: https://bioconductor.org/packages/release/bioc/html/RadioGx.html
